# Supplementary figures and images for: Rab5c-mediated endocytic trafficking regulates hematopoietic stem and progenitor cell development via Notch and AKT signaling
Source: PLoS Biol. 2020 Apr 10;18(4):e3000696. doi: 10.1371/journal.pbio.3000696 (PMC7176290; doi:10.1371/journal.pbio.3000696)

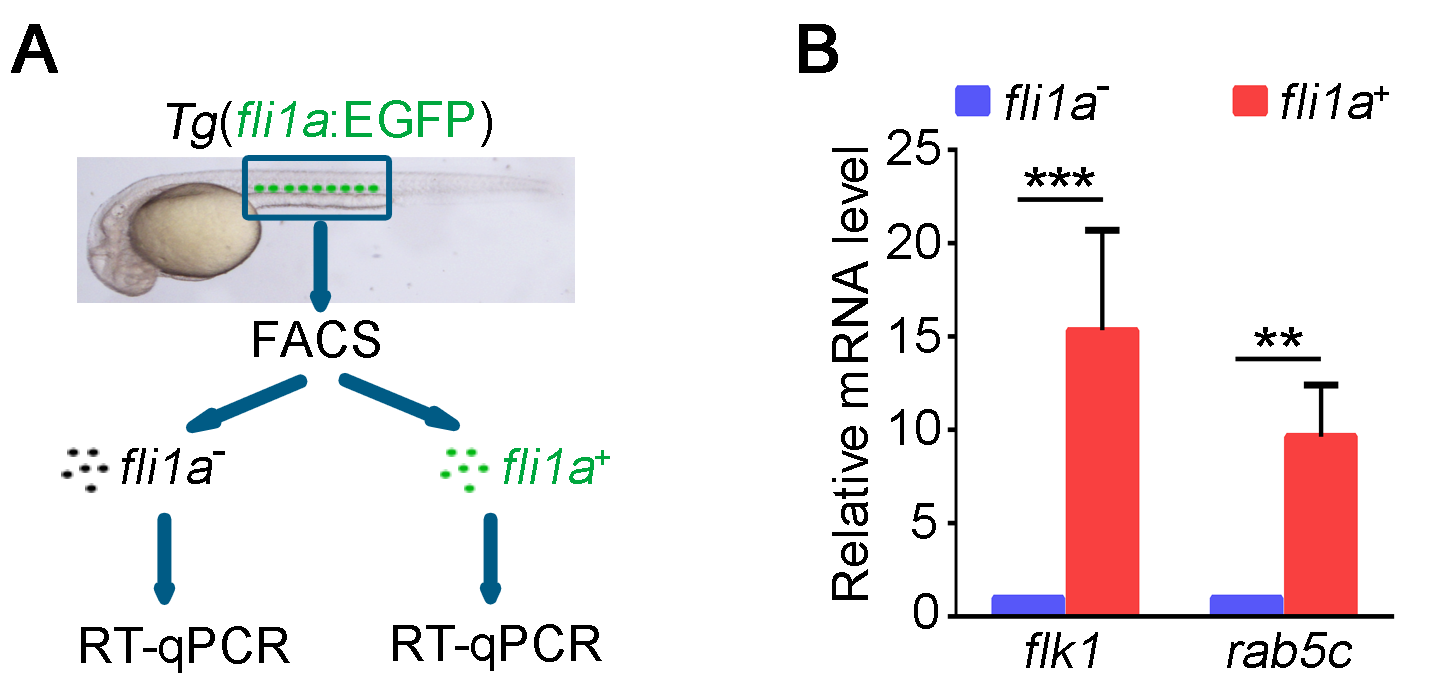

Supplement: S1 Fig — (A) Flowchart of FACS and qRT-PCR analysis of endothelial and nonendothelial cells in the trunk region from Tg(fli1a:EGFP) transgenic zebrafish embryos at 26 hpf. The trunk region in this transgenic line was dissected and dispersed into single cells for FACS and qRT-PCR. (B) qRT-PCR analysis of fli1a− and fli1a+ cells. The flk1 was used as positive control for endothelium specific expression gene. The expression of rab5c is enriched in fli1a+ ECs. P value was calculated by Student t test, **P < 0.01,***P < 0.001. The underlying data in this figure can be found in S1 Data. EC, endothelial cell; FACS, fluorescence-activated cell sorting; hpf, hours post fertilization; qPT-PCR, quantitative reverse-transcription PCR (TIF) [file pbio.3000696.s001.tif]

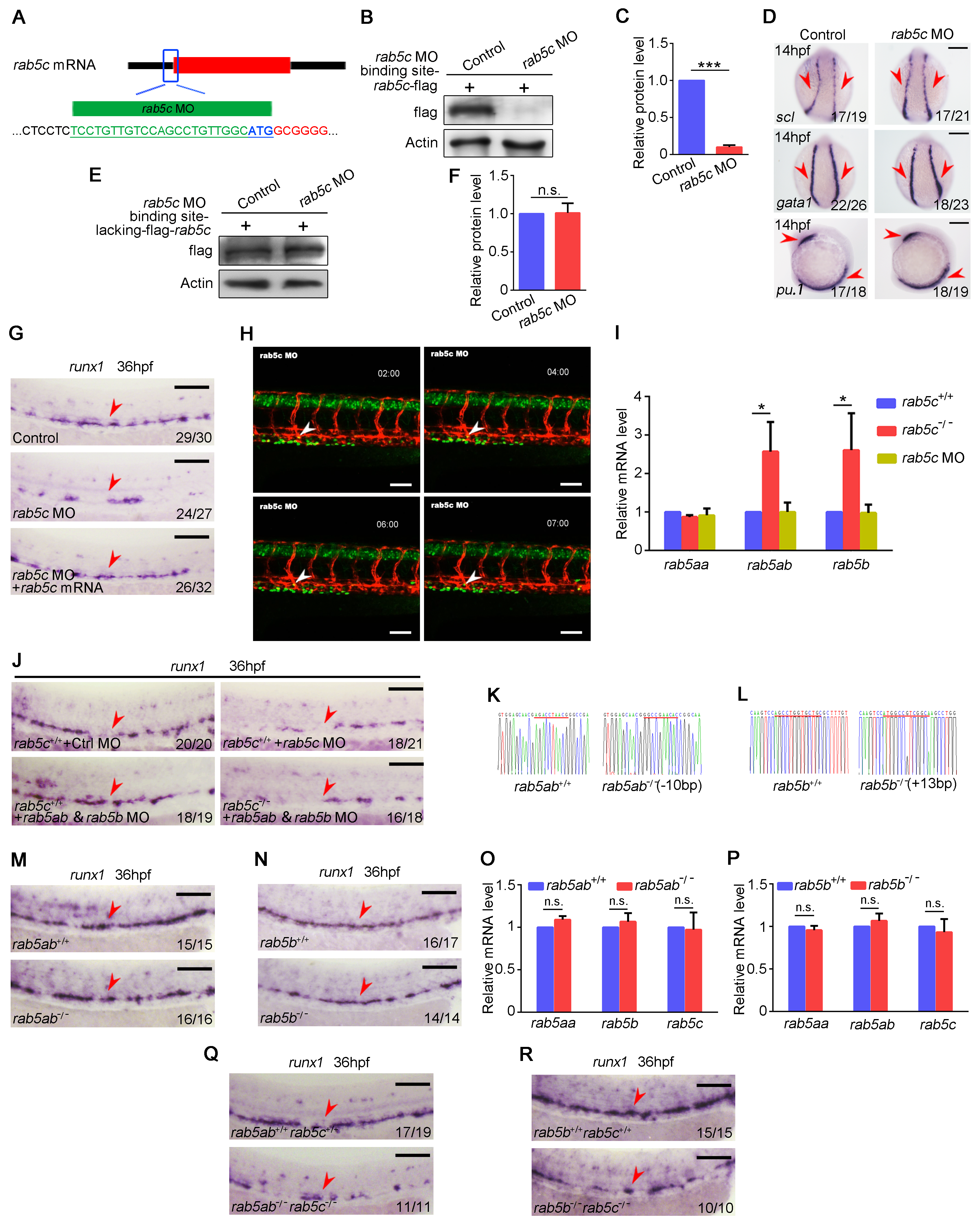

Supplement: S2 Fig — (A) Design of rab5c MO targeting the intersection of 5′UTR and CDS of rab5c mRNA for translation blocking. The start codon of rab5c is in blue. (B) KD efficiency of rab5c MO examined by WB. Flag-tagged mRNA containing the rab5c MO binding site and full-length rab5c CDS was co-injected with either control or rab5c MO into one-cell stage embryos. Rab5c-Flag was detected by anti-Flag antibody. (C) Quantification of protein level using gray analysis (Gel-Pro analyzer). Error bars, mean ± SD, ***P < 0.001. (D) Rab5c-deficiency does not impair primitive hematopoiesis. Expression of scl in hemangioblast, gata1 in red blood cells, and pu.1 in myeloid cells is not changed in rab5c morphants compared with control. This examination was carried out using WISH. The numbers below the WISH pictures mean number of embryos showing representative phenotype/total number of embryos. Scale bar, 100 μm. (E) Flag-tagged rab5c mRNA lacking the rab5c MO binding site was co-injected with either control or rab5c MO into one-cell stage embryos. The protein level was examined by WB. (F) Quantification of protein level using gray analysis (Gel-Pro analyzer). Error bars, mean ± SD. (G) HSPC rescue of rab5c morphants with rab5c mRNA. rab5c mRNA lacking the rab5c MO binding site can rescue the expression of HSPC marker runx1 in rab5c morphants. The red arrowheads denote HSPCs. Scale bar, 100 μm. (H) Snapshot in S4 Movie. Time-lapse imaging shows EHT process in rab5c morphants. The arrow denotes the cell undergoing EHT progress. Scale bar, 100 μm. (I) Relative mRNA level of other zebrafish Rab5 family genes rab5aa, rab5ab, rab5b in rab5c WT, mutant, and rab5c morphants at 26 hpf examined by qRT-PCR. Error bars, mean ± SD, *P < 0.05. (J) WISH results show that expression of runx1 is unchanged in low-dose of rab5ab and rab5b MOs co-injected rab5c WT embryos but is severely decreased in low-dose of MOs co-injected rab5c mutant embryos. Scale bar, 100 μm. (K) Generation of rab5ab mutant using the CRISPR [file pbio.3000696.s002.tif]

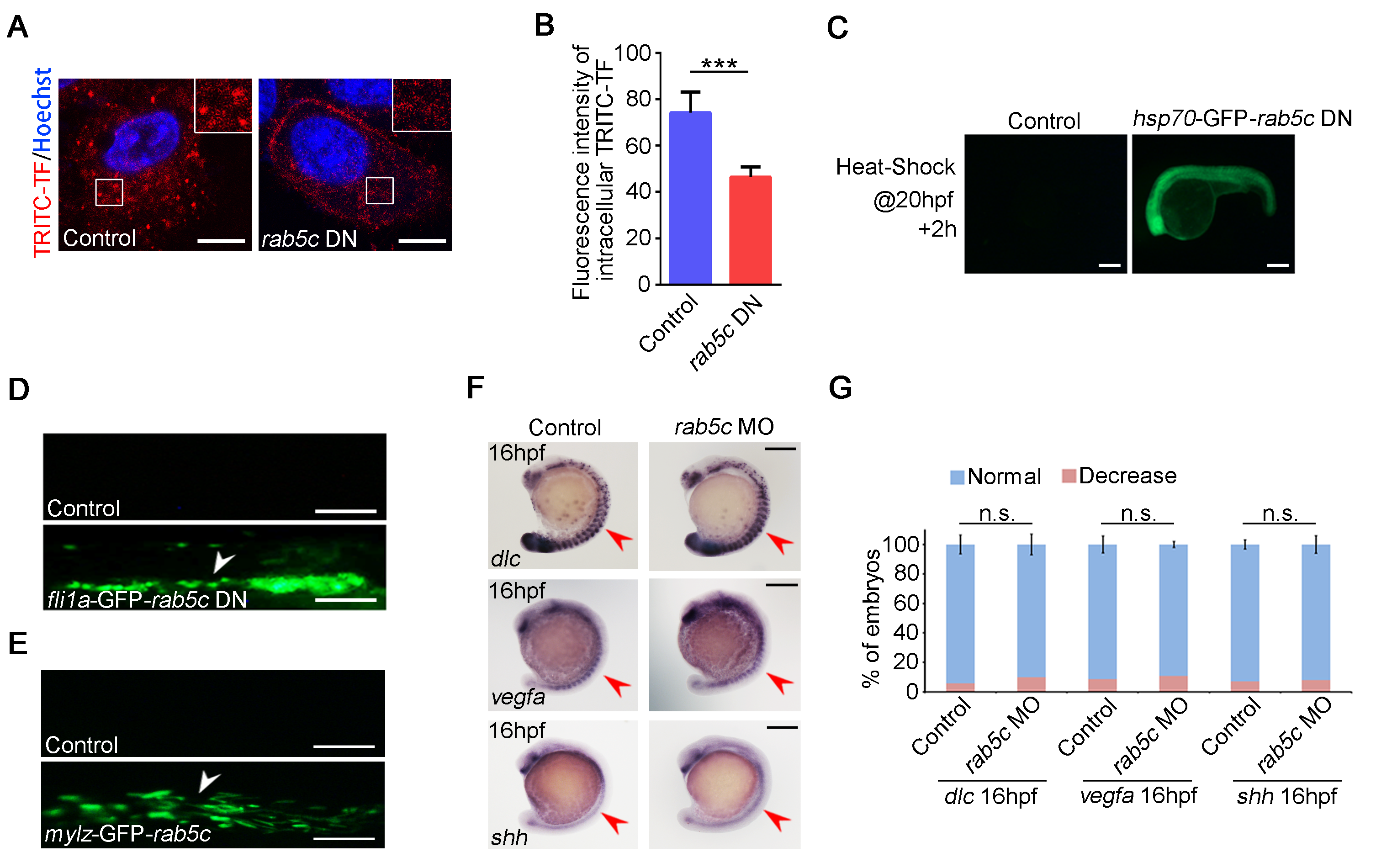

Supplement: S3 Fig — (A) TRITC-conjugated TF internalization assay in Hela cells transfected with empty pCS2 or pCS2-rab5c DN plasmids. Representative pictures were shown. Scale bar, 10 μm. (B) Quantitative fluorescence intensity of intracellular TRITC-TF in empty pCS2 or pCS2-rab5c DN transfected Hela cells, n = 8 cells for each group. Error bars, mean ± SD. P value was calculated by Student t test, ***P < 0.001. (C) Fluorescence microscope imaging shows that the GFP expression is detected by 2 hours post HS at 20 hpf in hsp70-GFP-rab5c DN group, but not in control. Scale bar, 200 μm. (D) Fluorescence microscope imaging shows that the GFP expression is detected in ECs of fli1a-GFP-rab5c DN group, but not in control. Scale bar, 200 μm. (E) Fluorescence microscope imaging shows that the GFP expression is detected in somitic cells of mylz-GFP-rab5c group but not in the control. Scale bar, 200 μm. (F) Expression of dlc, vegfa, and shh in control and rab5c morphants examined by WISH. Scale bars, 400 μm. (G) Statistical analysis of the WISH. Error bars, mean ± SD. The P values in this figure were calculated by Student t test. The underlying data in this figure can be found in S1 Data. DN, dominant-negative; EC, endothelial cell; GFP, green fluorescent protein; hpf, hours post fertilization; HS, heat shock; n.s., nonsignificant; TF, transferrin; TRITC, tetramethylrhodamine; WISH, whole-mount in situ hybridization (TIF) [file pbio.3000696.s003.tif]

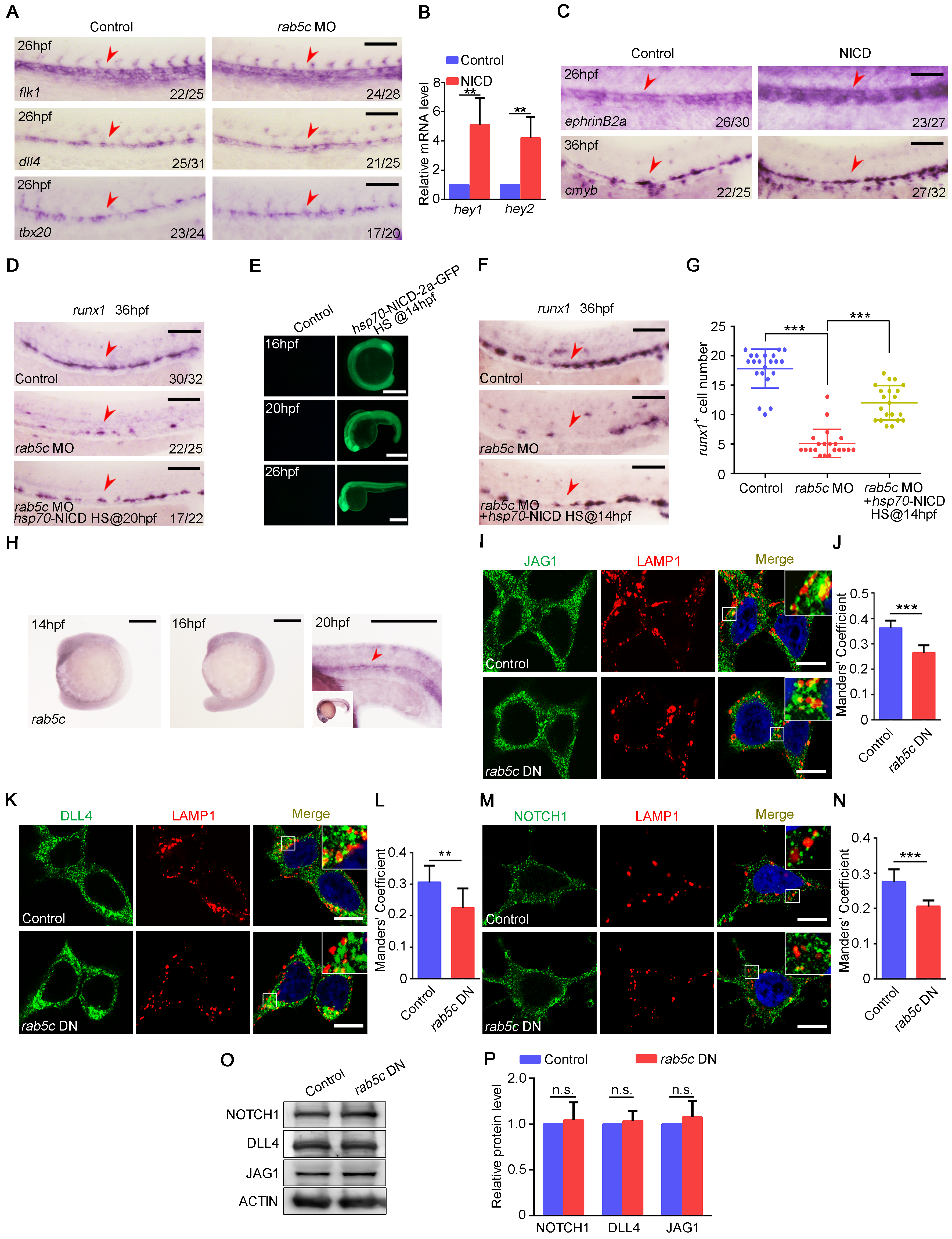

Supplement: S4 Fig — (A) Expression of panvascular gene flk1, Notch ligand gene dll4, and Notch-independent arterial marker tbx20 in the control and the rab5c morphants at 26 hpf examined by WISH. The numbers below the WISH pictures mean number of embryos showing representative phenotype/total number of embryos. Scale bar, 100 μm. (B) qRT-PCR results show that NICD overexpression leads to Notch downstream gene hey1 and hey2 up-regulation. NICD overexpression was carried out by hsp70-NICD HS at 20 hpf. (C) WISH results show that NICD overexpression leads to up-regulation of ephrinB2a positive ECs and cmyb-marked HSPCs. Scale bar, 100 μm. (D) WISH shows that runx1 expression in rab5c morphants is partially rescued by NICD overexpression through hsp70-NICD-2a-GFP HS at 20 hpf. Scale bar, 100 μm. (E) Fluorescence microscope imaging shows the GFP expression. Scale bar, 400 μm. (F) Expression of runx1 in control and rab5c morphants examined by WISH. Scale bars, 100 μm. (G) Quantification of the runx1+ cells. Error bars, mean ± SD, ***P < 0.001. (H) The expression pattern of rab5c during zebrafish early developmental stage examined by WISH. Scale bars, 400 μm. (I) Control empty plasmid or pCS2-rab5c DN transfected 293T cells were immunostained with antibodies against endogenous JAG1 (green) and LAMP1 (red). Scale bar, 10 μm. (J) Quantification of co-localization of JAG1 with LAMP1 using Manders’ coefficient (ImageJ). n = 14 cells. Error bars, mean ± SD, ***P < 0.001. (K) Control plasmid or pCS2-rab5c DN transfected 293T cells were immunostained with antibodies against endogenous DLL4 (green) and LAMP1 (red). Scale bar, 10 μm. (L) Quantification of co-localization of DLL4 with LAMP1 using Manders’ coefficient. n = 14 cells. Error bars, mean ± SD, **P < 0.01. (M) Control plasmid or pCS2-rab5c DN transfected 293T cells were immunostained with antibodies against endogenous NOTCH1 (green) and LAMP1 (red). Scale bar, 10 μm. (N) Quantification of co-localization of NOTCH1 with LAMP1 using Manders’ co [file pbio.3000696.s004.tif]

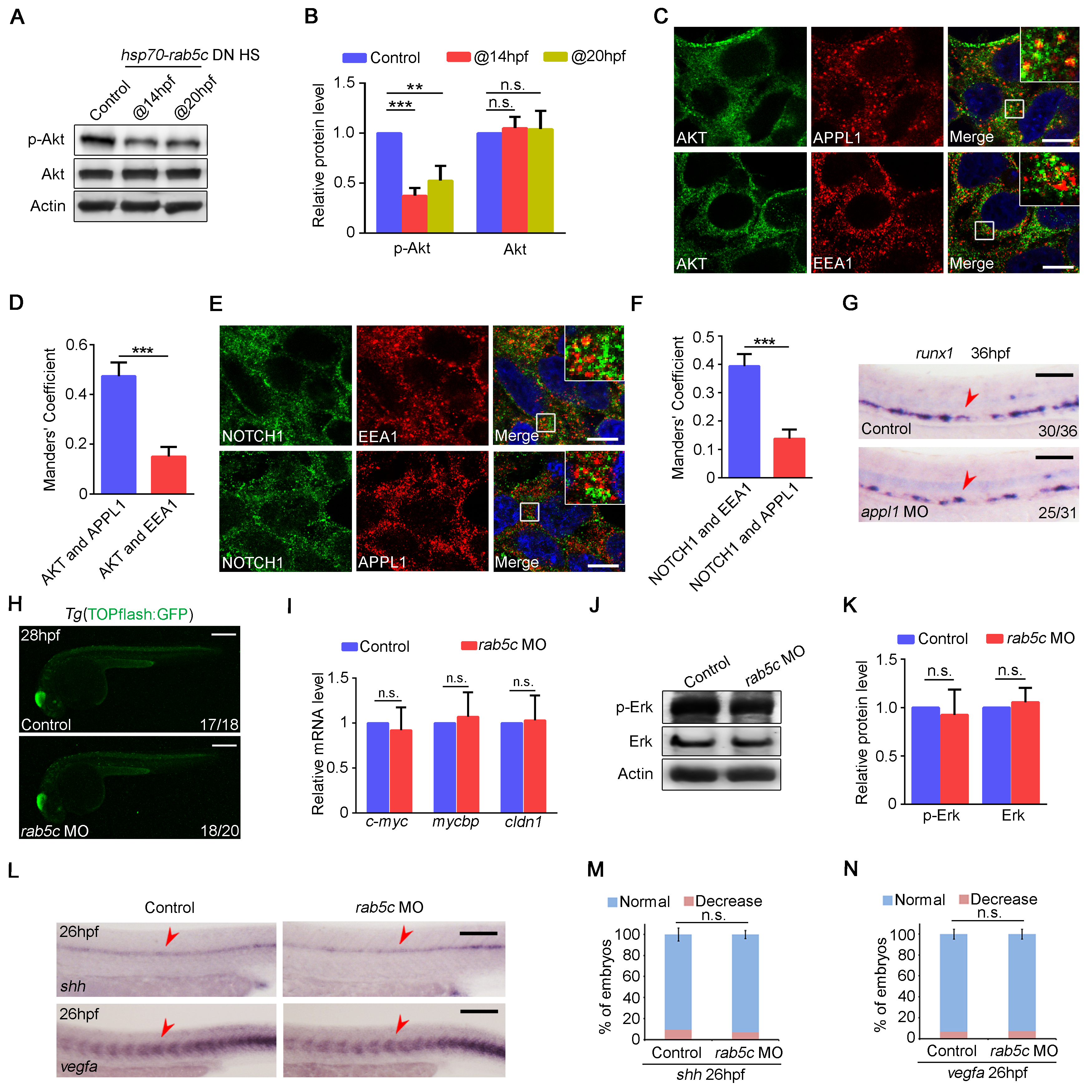

Supplement: S5 Fig — (A) Protein level of p-Akt and total Akt in control and Rab5c inhibition group at 26 hpf examined by WB. rab5c DN overexpression was carried out by hsp70-GFP-rab5c DN HS at 14 or 20 hpf. (B) Quantification of protein level using gray analysis (Gel-Pro analyzer). Error bars, mean ± SD, **P < 0.01, ***P < 0.001. (C) 293T cells were immunostained with antibodies against endogenous AKT (green), APPL1 (red), or EEA1 (red). The nucleus was stained with Hoechst. Scale bar, 10 μm. (D) Quantification of co-localization of target proteins using Manders’ coefficient (ImageJ). n = 14 cells. Error bars, mean ± SD, ***P < 0.001. (E) 293T cells were immunostained with antibodies against endogenous NOTCH1 (green), EEA1 (red), or APPL1 (red). The nucleus was stained with Hoechst. Scale bar, 10 μm. (F) Quantification of co-localization of target proteins using Manders’ coefficient (ImageJ). n = 14 cells. Error bars, mean ± SD, ***P < 0.001. (G) Expression of runx1 in control and appl1 morphants examined by WISH. Scale bar, 100 μm. The numbers below the WISH pictures mean: number of embryos showing representative phenotype/total number of embryos. (H) Examination of Wnt activity by Tg(TOPflash:GFP) line. Scale bar, 100 μm. (I) Examination of Wnt signaling downstream gene c-myc, mycbp, and cldn1 expression by qRT-PCR. Error bars, mean ± SD. (J) Protein level of Erk and p-Erk in control and rab5c morphants at 26 hpf. (K) Quantification of protein level using gray analysis (Gel-Pro analyzer). Error bars, mean ± SD. (L) Expression of shh and vegfa in control and rab5c morphants examined by WISH. Scale bars, 100 μm. (M) Statistical analysis of the WISH examining shh. Error bars, mean ± SD. (N) Statistical analysis of the WISH examining vegfa. Error bars, mean ± SD. The P values in this figure were calculated by Student t test. The underlying data in this figure can be found in S1 Data. DN, dominant-negative; hpf, hours post fertilization; HS, heat shock; n.s., nonsignificant; p-Akt, phosph [file pbio.3000696.s005.tif]

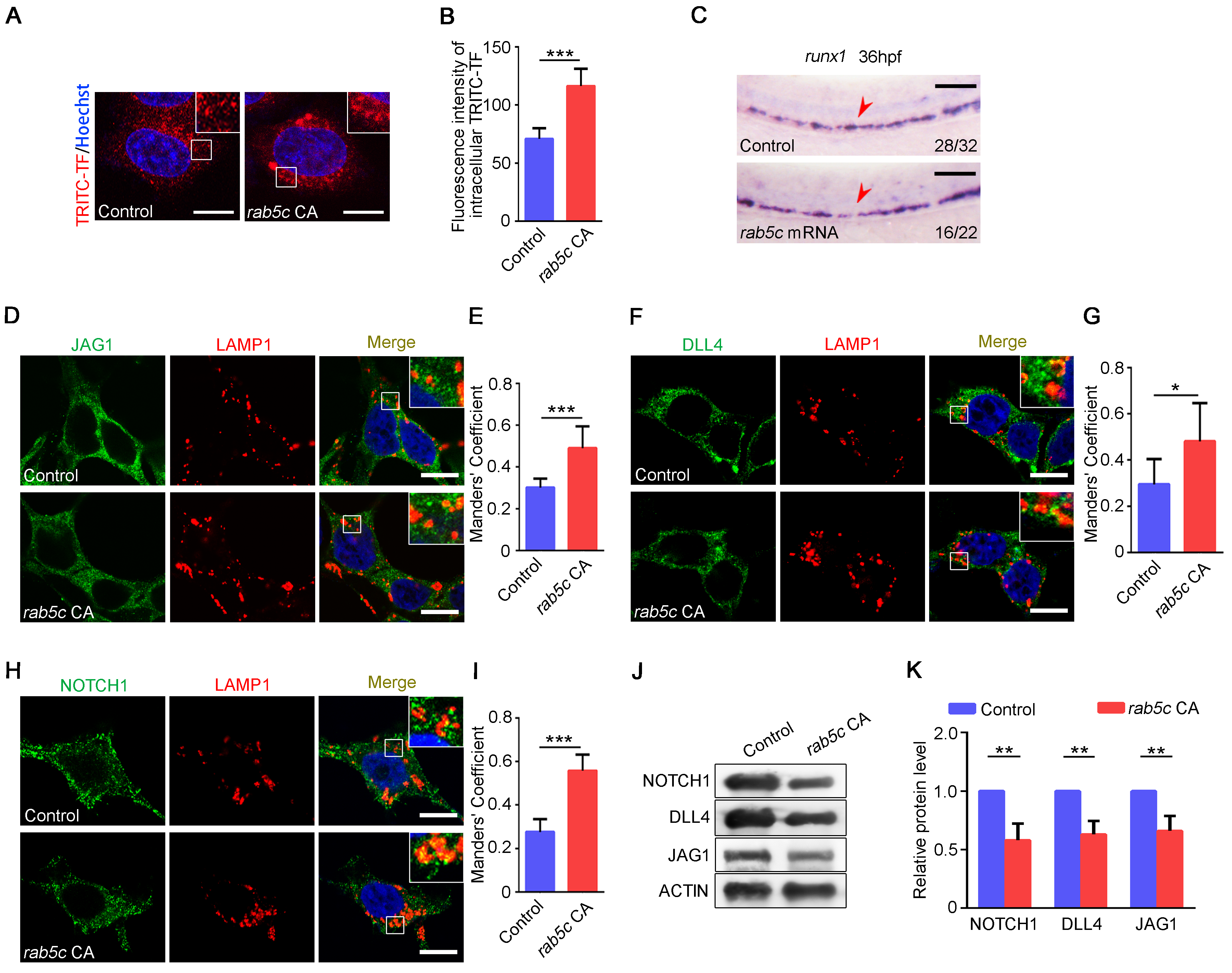

Supplement: S6 Fig — (A) TRITC-conjugated TF internalization assay in Hela cells transfected with control empty pCS2 or pCS2-rab5c CA plasmid. Representative pictures were shown. Scale bar, 10 μm. (B) Quantitative fluorescence intensity of intracellular TRITC-TF in control empty pCS2 and pCS2-rab5c CA transfected cells, n = 8 cells for each group. Error bars, mean ± SD. P value was calculated by Student t test, ***P < 0.001. (C) rab5c WT mRNA overexpression does not lead to HSPC production alteration. WISH results show that the expression of runx1 is not changed in rab5c WT mRNA overexpression group compared with control. The numbers below the WISH pictures mean: number of embryos showing representative phenotype/total number of embryos. Scale bar, 100 μm. (D) Control empty pCS2 or pCS2-rab5c CA transfected 293T cells were immunostained with antibodies against endogenous JAG1 (green) and LAMP1 (red). Scale bar, 10 μm. (E) Quantification of co-localization of JAG1 with LAMP1 using Manders’ coefficient (ImageJ). n = 14 cells. Error bars, mean ± SD, ***P < 0.001. (F) Control plasmid or pCS2-rab5c CA transfected 293T cells were immunostained with antibodies against endogenous DLL4 (green) and LAMP1 (red). Scale bar, 10 μm. (G) Quantification of co-localization of DLL4 with LAMP1 using Manders’ coefficient. n = 14 cells. Error bars, mean ± SD, *P < 0.05. (H) Control plasmid or pCS2-rab5c CA transfected 293T cells were immunostained with antibodies against endogenous NOTCH1 (green) and LAMP1 (red). Scale bar, 10 μm. (I) Quantification of co-localization of NOTCH1 with LAMP1 using Manders’ coefficient. n = 14 cells. Error bars, mean ± SD, ***P < 0.001. (J) Protein level of NOTCH1, DLL4, JAG1 in control empty pCS2 or pCS2-rab5c CA transfected 293T cells examined by WB. (K) Quantification of protein level using gray analysis (Gel-Pro analyzer). Error bars, mean ± SD, **P < 0.01. The P values in this figure were calculated by Student t test. The underlying data in this figure can be found in S1 D [file pbio.3000696.s006.tif]

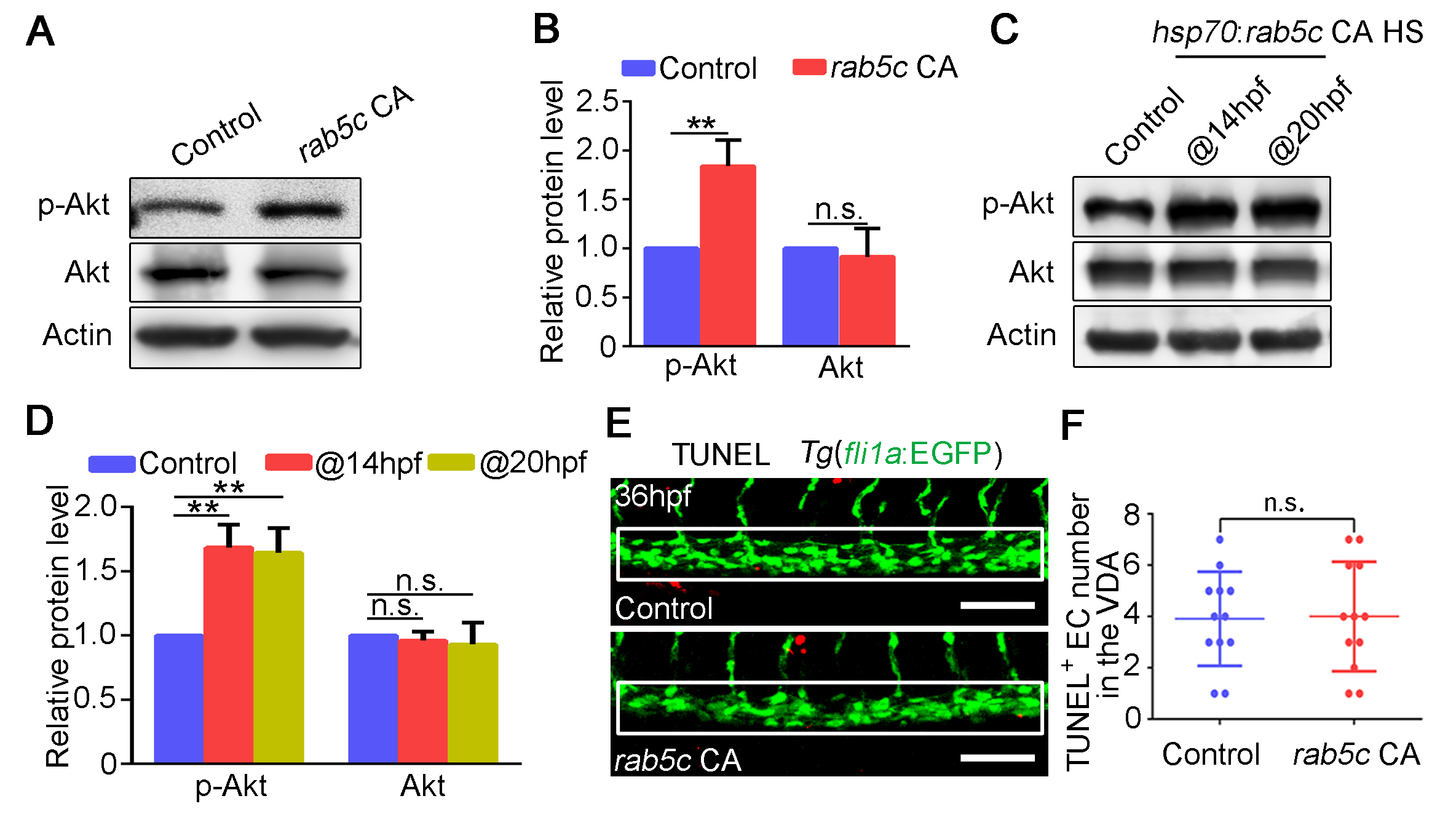

Supplement: S7 Fig — (A) Protein level of p-Akt and total Akt in control and rab5c CA mRNA injected embryos examined by WB. (B) Quantification of protein level using gray analysis (Gel-Pro analyzer). Error bars, mean ± SD, **P < 0.01. (C) Protein level of p-Akt and total Akt in control and Rab5c CA group examined by WB. hsp70-rab5c CA injected embryos were heat-shocked at 14 hpf or 20 hpf for Rab5c CA overexpression. (D) Quantification of protein level using gray analysis. Error bars, mean ± SD, **P < 0.01. (E) TUNEL assay shows the apoptotic fli1a+ (yellow) cells in the VDA region (box area) of control and rab5c CA mRNA overexpression group. Scale bar, 100 μm. (F) Quantification of TUNEL+ ECs. Error bars, mean ± SD. The P values in this figure were calculated by Student t test. The underlying data in this figure can be found in S1 Data. CA, constitutively active; EC, endothelial cell; hpf, hours post fertilization; HSPC, hematopoietic stem and progenitor cell; n.s., nonsignificant; p-Akt, phosphorylated Akt; TUNEL, terminal-deoxynucleoitidyl transferase mediated nick end labeling; VDA, ventral wall of the dorsal aorta; WB, western blot (TIF) [file pbio.3000696.s007.tif]

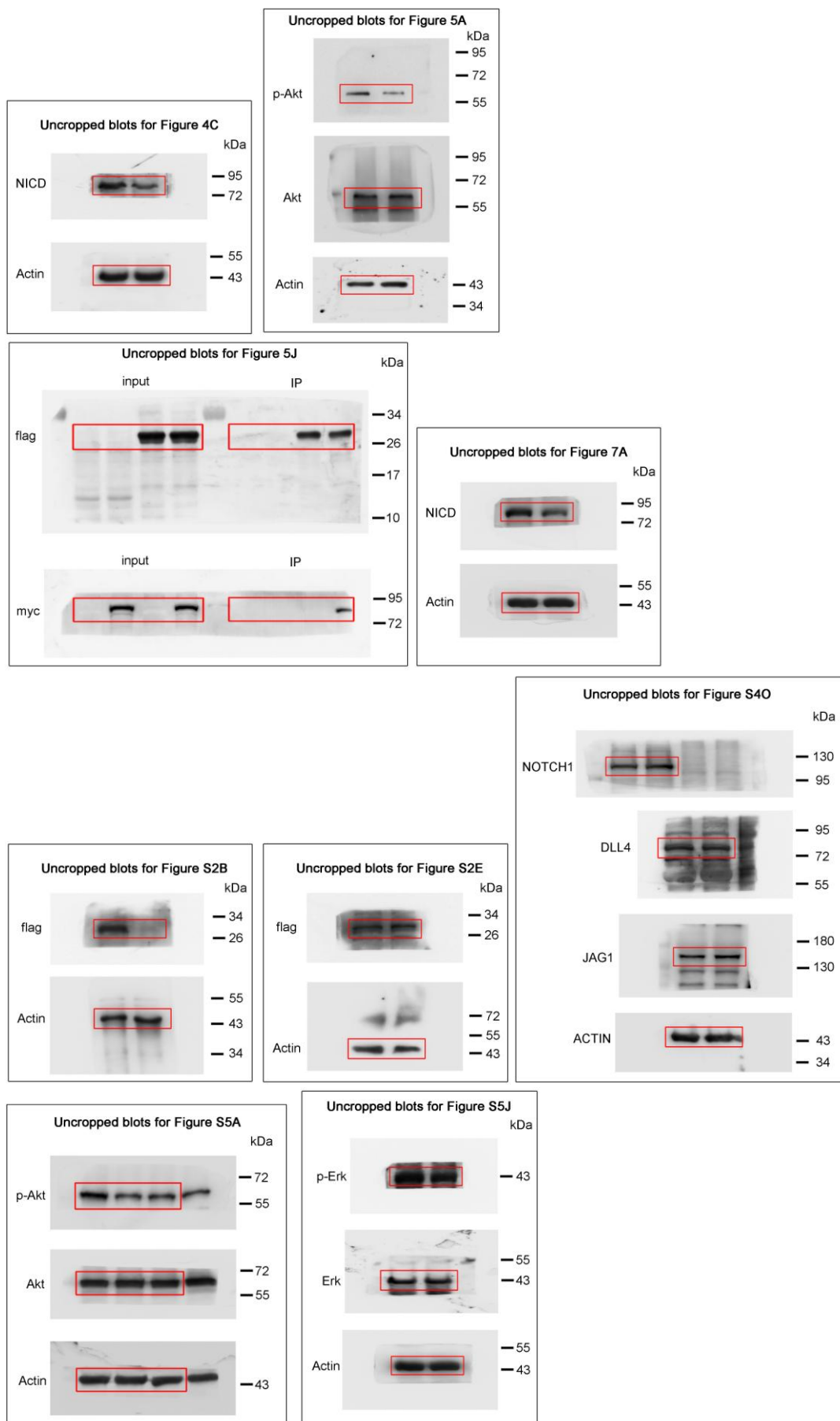

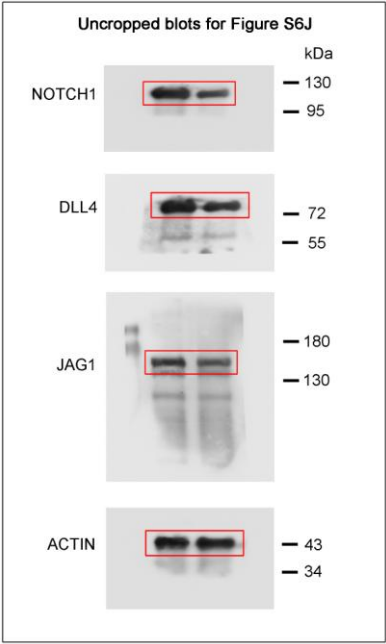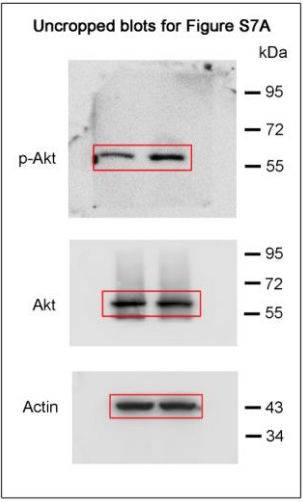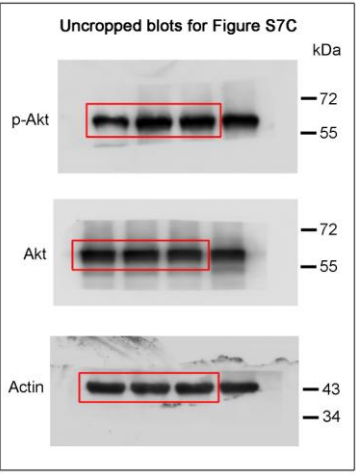

Supplement: S1 Raw Images — (PDF) [file pbio.3000696.s008.pdf]
