# Supplementary material for: Rab5c-mediated endocytic trafficking regulates hematopoietic stem and progenitor cell development via Notch and AKT signaling
Source: PLoS Biol. 2020 Apr 10;18(4):e3000696. doi: 10.1371/journal.pbio.3000696 (PMC7176290; doi:10.1371/journal.pbio.3000696)
Supplement: S1 Table — (DOC) [file pbio.3000696.s016.doc]

**S1 Table**

**The primers for point mutation e**xperiments.

| primers | Sequence |
| --- | --- |
| *rab5c*-S36N-F | 5’- AGTCTGCAGTAGGCAAGAACAGCCTGGTGCTGCGCTTCGTCAAAGGCCA-3’ |
| *rab5c*-S36N-R | 5’- AGCGCAGCACCAGGCTGTTCTTGCCTACTGCAGACTCTCCCAGCAACA-3’ |
| *rab5c*-Q81L-F | 5’- TTGGGACACGGCCGGACTGGAGCGGTATCACAGTTTGGCCCCTATGT-3’ |
| *rab5c*-Q81L-R | 5’- AACTGTGATACCGCTCCAGTCCGGCCGTGTCCCAAATCTCAAACTT-3’ |
