# Supplementary material for: Rab5c-mediated endocytic trafficking regulates hematopoietic stem and progenitor cell development via Notch and AKT signaling
Source: PLoS Biol. 2020 Apr 10;18(4):e3000696. doi: 10.1371/journal.pbio.3000696 (PMC7176290; doi:10.1371/journal.pbio.3000696)
Supplement: S2 Table — gRNA, guide RNA; PAM, protospacer adjacent motif (DOC) [file pbio.3000696.s017.doc]

**S2 Table**

**The gRNA target and PAM sequences**.

| gRNAs | Target and PAM sequence |
| --- | --- |
| *rab5c* gRNA | 5’-GGTGGACCAGCGCGGACCAA CGG-3’ |
| *rab5ab* gRNA | 5’-GGTGGAGCAACGAGACCTAA CGG-3’ |
| *rab5b* gRNA | 5’-GGCCGTCGGCAAGTCCAGCC TGG-3’ |
