# Supplementary material for: Rab5c-mediated endocytic trafficking regulates hematopoietic stem and progenitor cell development via Notch and AKT signaling
Source: PLoS Biol. 2020 Apr 10;18(4):e3000696. doi: 10.1371/journal.pbio.3000696 (PMC7176290; doi:10.1371/journal.pbio.3000696)
Supplement: S3 Table — qRT-PCR, quantitative reverse-transcription PCR (DOC) [file pbio.3000696.s018.doc]

**S3 Table**

**The primers for qRT-PCR and mutant screening**.

| primers | Sequence | Used in Experiment |
| --- | --- | --- |
| *rab5aa* F | 5’- TTGAGATCTGGGACACAGCT-3’ | qRT-PCR |
| *rab5aa* R | 5’- GCTAGCCTGCCTCTGAAGCT-3’ | qRT-PCR |
| *rab5ab* F | 5’- GTCAGTTCAAATTAGTGCTGCT-3’ | qRT-PCR |
| *rab5ab* R | 5’- ACCGTTGTGTCATCCAGACA-3’ | qRT-PCR |
| *rab5b* F | 5’- CTGGTGCTGCGCTTTGTGAA-3’ | qRT-PCR |
| *rab5b* R | 5’- AGCCAAGCTGTGGTAGCGCT-3’ | qRT-PCR |
| *rab5c* F | 5’- CTGCCAGTTTAAGCTTGTGT-3’ | qRT-PCR |
| *rab5c* R | 5’- CGTATCGTCCAAGCAGAGTGT-3’ | qRT-PCR |
| *gapdh* F | 5’- GTCTTCACTACTATTGAGAAGGC-3’ | qRT-PCR |
| *gapdh* R | 5’- GCAGTTGGTGGTGCAGGAGGCA-3’ | qRT-PCR |
| *hey1* F | 5’- TCGAAGTGGAGAAGGAGAGTG-3’ | qRT-PCR |
| *hey1* R | 5’- TCCCTCCGGCGCTTCTCGAT-3’ | qRT-PCR |
| *hey2* F | 5’- GAAGCGGCCCTGTGAGGACA-3’ | qRT-PCR |
| *hey2* R | 5’- CTTTCTGGCCATGACTTGGGA-3’ | qRT-PCR |
| *flk1* F | 5’- CACACTCATTTGCAGAGGATC-3’ | qRT-PCR |
| *flk1* R | 5’- GTGACTGCATGAGTAGATGCC-3’ | qRT-PCR |
| *myc* F | 5’- TCCGCCGCTGCCAAGTTGGAG-3’ | qRT-PCR |
| *myc* R | 5’-GAAGACCACAGAGGGATCGATG-3’ | qRT-PCR |
| *mycbp* F | 5’-CTCGACAGCCTGACCAACGTC-3’ | qRT-PCR |
| *mycbp* R | 5’-TTCTGCTGCAGTGTGTTCAG-3’ | qRT-PCR |
| *cldn1* F | 5’-CTGGGTCTGCTGGGTCTGATC-3’ | qRT-PCR |
| *cldn1* R | 5’-TGCACTGCAGCTGGCCGGTG-3’ | qRT-PCR |
| *rab5c-*mu-F | 5’- AAGCTTGTCGAATCCATCGA-3’ | Mutant Screening |
| *rab5c-*mu-R | 5’ -CTTTGACGAAGCGCAGCACCA-3’ | Mutant Screening |
| *rab5ab-*mu-F | 5’ -CTGGTAGTGACCGTCTTGAC-3’ | Mutant Screening |
| *rab5ab-*mu-R | 5’ -CAAAGCGCAGCACTAGACTCGA-3’ | Mutant Screening |
| *rab5b-*mu-F | 5’ - CCGCAGACCAAGATCTGCCAGT-3’ | Mutant Screening |
| *rab5b-*mu-R | 5’ - CCAATCCATATGAACTCAAATCA-3’ | Mutant Screening |
