# Supplementary material for: Rab5c-mediated endocytic trafficking regulates hematopoietic stem and progenitor cell development via Notch and AKT signaling
Source: PLoS Biol. 2020 Apr 10;18(4):e3000696. doi: 10.1371/journal.pbio.3000696 (PMC7176290; doi:10.1371/journal.pbio.3000696)
Supplement: Attachment — Submitted filename: PBP Response_R1.doc [file pbio.3000696.s020.doc]

**Revision summary and Point-by-Point Response to Reviewers**

1. **Summary of major comments from the reviewers.**

Based on the requests and comments from the reviewers, we have performed further analyses and additional experiments. Our major revision and new supporting data are summarized and listed in the following Table 1.

**Table 1. Revision summary and new supporting data to address major comments from the reviewers.**

| **Questions** | | **Reviewers** | **Clarification on the original submission data and New data supporting** |
| --- | --- | --- | --- |
| 1 | Does Rab5c co-immunoprecipitates with Notch1 or with Akt? | 1#: Q1 | To examine whether Rab5c co-immunoprecipitates with Notch1 or with Akt, we performed co- IP by flag-tagged Rab5c and anti-flag antibody-bead mixture in 293T cells. The results showed that Rab5c not only co-immunoprecipitated with Notch1, but also co-immunoprecipitated with Akt (Response New Figure 1, Fig.1A-D). |
| 2 | Could they show some data in the endothelium of zebrafish? Their conclusions are sometimes too strong considering that they never address the endocytic detection in zebrafish. For example in Page 10, line 226: we concluded that Rab5c is essential for HE specification through endocytic trafficking-regulated Notch signaling. | 1#: Q3 | We tried to check the localization of Notch ligand and receptor, Eea1 and Appl1 in the endothelium of zebrafish by IF. However, the antibodies, which worked well in human cell lines, did not specifically recognize zebrafish endogenous target proteins (Response New Figure 2, Fig.2A). Besides, in our zebrafish endothelium imaging experiments, the maximum magnification was 200×, which could resolve different types of cells but not subcellular organelles or complexes. We have toned down our conclusions accordingly in the revised manuscript (see revised manuscript, Page 10, line 246-247: Taken together, we speculate that Rab5c is important for HE specification likely through endocytic trafficking-regulated Notch signaling). |
| 3 | The authors colocalize endosomes with Notch1, Jag1 and Dll4. Are they together in the same endosomes? Is Notch1 co-localizing with any of the ligands? Is the Notch1 antibody recognizing the intracellular or the extracellular domain? The interpretation would be different depending on what kind of Notch are they detecting. | 1#: Q4 | We performed IF experiment in 293T cell. The result (Response New Figure 3, Fig.3A and B) showed that Notch receptor Notch1 and the Notch ligand Dll4 were almost not together in the same endosome. In addition, the result showed that Notch1 barely colocalized with Dll4. This result may be due to the character of the Notch1 antibody we used. This Notch1 antibody specifically recognizes the Notch transmembrane subunit (NTM) region, which consists of a short extracellular juxtamembrane peptide, a transmembrane sequence and the intracellular domain (NICD). The Notch ligand mainly binds with extracellular Notch subunit (ECN) but not NTM. The early endosome localization of NTM is a required step for NICD release and Notch signal activation in normal circumstances (1). |
| 4 | Do the authors suggest that Notch endosomes are only interacting with EEA1 and LAMP1? Could they show negative controls? they could test colocalization of Notch members with Appl1 and Akt with EEA1. If the authors are correct, those proteins should not co-localize. | 1#: Q7 | We performed IF experiment in 293T cell line. The result (Response New Figure 4, Fig.4A and B, also see revised manuscript Fig. S5 D and E) showed that the Notch receptor Notch1 barely colocalized with APPL1. In addition, AKT mainly colocalized with APPL1 but not EEA1 (Response New Figure 4, Fig.4C and D, also see revised manuscript Fig. S5 B and C). These results suggest that Notch signal is mainly regulated by endosome labeled by EEA1, and AKT signal is mainly regulated by endosome labeled by APPL1. |
| 5 | Western blots in Fig. 3C, 4A, 4G and 6A shows a single experiment. It would be helpful to provide quantitative analysis of western blot data from several independent experiments. | 2#: Q1 | We performed quantitative analysis of all these western blot data from several independent experiments and presented statistical results in the revised manuscript (see revised manuscript, Fig. 4D, 5B,5K,7B). |
| 6 | It has been previously shown, that Rab5c is a key regulator of trafficking of the CD93/Multimerin/β1-integrin complex in endothelial cell adhesion and migration. Furthermore, another recent study demonstrated that another small GTPase of the Ras-related protein family, Rap1b, promotes notch-mediated hemogenic endothelium specification of PLPM cells during HSPC specification by enhancing integrin-mediated cell adhesion. The authors should further investigate and comment on these recent findings in the context of their study. | 3#: Q2 | Both of CD93/Multimerin/β1-integrin complex and Rap1b are associated with β1-integrin activation (2, 3), which has been demonstrated to facilitate Notch signaling during early somitic wave (15-17 hpf) (3, 4). Previous studies showed that heat-shock induction of NICD at 14 hpf can rescue HSPC defects induced by repression of early somitic Notch signaling, however, heat-shock just 2 h later (about 16 hpf) can not (4, 5). In our study, induction of Notch signaling by heat-shock-induced overexpression of NICD at 20 hpf can rescue HSPC defects in Rab5c-deficiency embryos, suggesting that Rab5c regulates Notch during the late endothelial wave (after 20 hpf), but not early somitic wave. Furthermore, we examined the expression pattern of *rab5c* during zebrafish early developmental stage by whole-mount in situ hybridization (WISH). The result (Response New Figure 5, Fig. 5A, also see revised manuscript Fig.S4H) showed that *rab5c* was almost not expressed at 14 hpf or 16 hpf, and was expressed specifically in endothelial cells at 20 hpf. Taken together, we propose that Rab5c may not be involved in integrin-mediated somitic wave regulation. We have added more discussions on these recent findings in the revised manuscript (see revised manuscript, Page 15-16, line 391-402). |
| 7 | They should also consider performing early NICD induction at 14 hpf and analysis of sclerotome markers to further confirm that this wave is not involved. Additionally, they should discuss in the text why they perform these different experiments here (in regard to the two different waves, somitic and endothelial, during HSPC development). | 3#: Q3 | To check whether Rab5c-deficiency impairs somitic wave of HSPC specification, we examined the expression of sclerotome markers by WISH. The results (Response New Figure 6, Fig. 6A and B, also see revised manuscript Fig.S3 F and G) showed that expression of *dlc*, *vegfa* and *shh* was not changed in Rab5c-dificiency embryos at 16 hpf, suggesting that Rab5c is not involved in somitic wave of HSPC specification. To perform early NICD induction at 14 hpf, we utilized *hsp70*-mediated heat-shock (HS) inducible NICD-2a-GFP overexpression system. Strong GFP expression was detected from 2 hours post heat-shock at 14 hpf (Response New Figure 7, Fig. 7A, also see revised manuscript Fig. S4E), to at least 20 hours post heat-shock. Notably, the GFP expression level at 20 or 26 hpf was comparable to that at 16 hpf (Response New Figure 7, Fig.7A, also see revised manuscript Fig. S4E). The WISH results showed that the HSPC defect in Rab5c-dificiency embryos could be rescued by the heat-shock induction of NICD at 14 hpf (Response New Figure 7, Fig.7B and C, also see revised manuscript Fig. S4 F and G). Although the heat-shock induction of Notch at 14 hpf provides a rescue, this induction is sustained throughout the endothelial wave (after 20 hpf). Previous studies showed that heat-shock induction of NICD at 14 hpf can rescue HSPC defects induced by repression of early somitic Notch signaling, however, heat-shock just 2 h later can not (4, 5). Together with that the heat-shock induction of NICD at 20 hpf rescuing the HSPC defect induced by Rab5c-dificiency, we propose that Rab5c regulates HSPC development at the endothelial wave. We will add more discussions in the text to address why we perform these different experiments (see revised manuscript, Page 9 line 208-212). |
|  | The authors show rab5c expression in endothelial cells at 26 hpf. If it is indeed responsible for notch pathway activation, it should already be present at earlier time-points between 16 and 20 hpf. Please explain/comment. | 3#: Q4 | We examined the expression pattern of rab5c during zebrafish early developmental stage by WISH. The result (Response New Figure 5, Fig. 5A,also see revised manuscript Fig.S4H) showed that rab5c was almost not expressed at 14 hpf or 16 hpf, and was specifically expressed in endothelial cells at 20 hpf. |
|  | Endothelial specific NICD-OE instead of global induction via HS would be better for the Notch rescue experiments in Figure 3 and 6. | 3#: Q5 | We performed endothelial cell (EC)-specific NICD-OE driven by *fli1a* promoter as previously described (6). The WISH results showed that the HSPC defect in Rab5c-dificiency embryos could be rescued by the EC-specific NICD-OE (Response New Figure 8, Fig.8A and B, also see revised manuscript Fig.4M and N). In addition, EC-specific NICD-OE also could rescue the HSPC defect induced by Rab5c CA overexpression (Response New Figure 9, Fig.9A and B, also see revised manuscript Fig.7I and J). We have used these new results to replace the HS-NICD data in the main text. |
|  | What about other pathways that regulate similar steps of HSPC development (e.g. Wnt, Erk signaling, Shh, Vegf signaling)? How do they relate to the presented pathway? | 3#: Q8 | The data in our original manuscript showed that Wnt and Erk signalings were not affected, and *flk1* expression was normal (data in original manuscript, now see revised manuscript Fig.S4A, Fig. S5 G-I), in Rab5c-dificiency embryos. We further examined the expression of *shh* and *vegfa* in control and Rab5c deficiency embryos. The WISH results showed that the expression of *shh* and *vegfa* at 16 or 26 hpf was not changed in Rab5c-dificiency embryos compared with control (Response New Figure 6, Fig.6A and B; Response New Figure 10, Fig.10A-C, also see revised manuscript Fig. S3 F and G, Fig.S5 J-L), suggesting that Shh and Vegf signalings might not be involved in Rab5c-dificiency induced HSPC defect. However, the relationship between these signaling pathways in Rab5c-regulated HSPC development awaits further investigation. |
|  | Please explain the western blot data shown in Supplementary Figure S2B. If wt mRNA was co-injected with the MO, shouldn't there be a rescue of the protein expression and no apparent down-regulation? Is there a specific antibody that can alternatively be used to show that indeed the protein is not there? | 3#: Q11 | This WT *rab5c* mRNA used in MO effectiveness test had the *rab5c* MO binding site. The MO could inhibit the translation of exogenous mRNA with its binding site (7). Previously, we performed the rescue experiment by overexpression of *rab5c* mRNA lacking the MO binding site. The western blot result showed that the translation of *rab5c* mRNA lacking the MO binding site was not inhibited by *rab5c* MO injection (Response New Figure 11, Fig.11A and B, also see revised manuscript Fig. S2 E and F). |
|  | What does relatively normal mean for their primitive blood phenotypes in Figure S2C? Are primitive myeloid cells affected? | 3#: Q15 | In the original manuscript, the primitive erythroid cells production was examined and the results showed normal primitive erythroid cells production in Rab5c-deficiency embryos. However, the data of primitive myeloid cells production was not presented. We further examined the primitive myeloid cells production by WISH. The results showed that the primitive myeloid cells production was also normal in Rab5c-deficiency embryos (Response New Figure 12, Fig.12A and B, also see revised manuscript Fig. S2D). Then we rephrased the statement to claim that primitive hematopoiesis was normal in Rab5c-deficiency embryos. |

**2. Point-by-point responses to reviewers**

Reviewer #1: This is an elegant work by Liu and colleagues that focus in the role of endosomal pathways in EHT. They found Rab5c in an RNA-seq screen of hemogenic specific genes. They have performed many the genetic controls and strategies by using knockdown, dominant negative or constitutively active forms, but also knockout of all Rab5 members (since. Rab5c alone was compensated). They found that depletion of Rab5c leads to a defect in definitive hematopoietic stem and progenitor cells and derivatives. Molecularly they found that Notch pathway is affected by checking the levels of N1IC and a Notch-fluorescent reporter. The levels of Notch are highly regulated by endosomes thus it is not surprising that an early endosome component affects this pathway. However, this is the first time that this specific interaction is reported.

The effect on the early hematopoietic development of Rab5c is convincing, and the relationship with Notch and AKT as well, however some questions remain about the mechanisms that could be further addressed.

Does Rab5c co-immunoprecipitates with Notch1 or with Akt?

**Response 1**: Thanks for this thoughtful comment. To examine whether Rab5c co-immunoprecipitates with Notch1 or with Akt, we performed co-immunoprecipitation (IP) by flag-tagged Rab5c and anti-flag beads in cell line. The results showed that Rab5c not only co-immunoprecipitated with Notch1, but also co-immunoprecipitated with Akt (Response New Figure 1, Fig.1A-D).

Response New Figure 1 (A) 293T cells were transfected with flag-tagged Rab5c constructs. Cell lysate was subjected to immunoprecipitation (IP) using anti-flag beads. The flag-Rab5c and endogenous NOTCH1 were analyzed by western blot. (B) Quantification of protein level using gray analysis (Gel-Pro analyzer). The ratios of NOTCH1 co-IP relative to input were calculated. Two replicates, Error bars, mean ± s.d, ****P* < 0.001. (C) 293T cells were transfected with flag-tagged Rab5c constructs. Cell lysate was subjected to IP using anti-flag beads. The flag-Rab5c and endogenous AKT were analyzed by western blot. (D) Quantification of protein level using gray analysis (Gel-Pro analyzer). The ratios of AKT co-IP relative to input were calculated. Two replicates, Error bars, mean ± s.d, ****P* < 0.001.

Is this early endosome localization of Notch a required step for activation of Notch, for Notch receptor recycling or for Notch degradation in lysosomes? The authors should address this question more directly.

**Response 2**: Thanks for these questions. Previous studies (1, 8) have been cited in our original manuscript for explaining that early endosome localization of Notch was a required step for activation of Notch in normal circumstances. Our data showed that Rab5c-deficiency reduced early endosome localization of Notch, further leading to Notch signaling defect. Our results supported this previous view. However, on the other hand, our data showed that overexpression of Rab5c constitutively active (CA) form, but not wild type (WT) form, resulted in increased Notch degradation in lysosomes. The Q81L amino acid mutation in the Rab5c CA affected GTPase activity and led to excessive endocytic trafficking of Notch. Rab5c CA likely led to a special circumstance in which largely increased early endosome localization of Notch enhanced endolysosomal trafficking of Notch. We have added more direct discussions on these questions in the revised manuscript (see revised manuscript, Page 16, line 407-415).

In addition, they only show data of co-localization in cell lines (293T or Hela), could they show some data in the endothelium of zebrafish? Their conclusions are sometimes too strong considering that they never address the endocytic detection in zebrafish. For example in Page 10, line 226:  we concluded that Rab5c is essential for HE specification through endocytic trafficking-regulated Notch signaling.

**Response 3**: Thanks for these questions. We tried to check the localization of Notch ligand and receptor, Eea1 and Appl1 in the endothelium of zebrafish by immunofluorescence (IF). However, the antibodies, which worked well in human cell lines, did not specifically recognize zebrafish endogenous target proteins (Response New Figure 2, Fig.2A). Besides, in our zebrafish endothelium imaging experiments (such as Fig.2F or Movie S3,S4 in revised manuscript) the maximum magnification was 200×, which could resolve different types of cells but not subcellular organelles or complexes. In comparison, the observation of magnification of 1000× in cell lines was able to resolve subcellular organelles or complexes. We agree with the reviewer’s concern about these overstated conclusions. We have toned down our conclusions accordingly in the revised manuscript (see revised manuscript, Page 10, line 246-247: Taken together, we speculate that Rab5c is important for HE specification likely through endocytic trafficking-regulated Notch signaling).

Response New Figure 2 (A) IF was carried out to detect zebrafish endogenous Jag1, Dll4, Notch1, Appl1 and Eea1 by respective antibodies. Confocal imaging showing that these antibodies do not recognize zebrafish endogenous target proteins. Scale bar, 400 μm.

The authors colocalize endosomes with Notch1, Jag1 and Dll4. Are they together in the same endosomes? Is Notch1 co-localizing with any of the ligands? Is the Notch1 antibody is recognizing the intracellular or the extracellular domain? The interpretation would be different depending on what kind of Notch are they detecting.

**Response 4**: Thanks for this thoughtful comment. To answer this question, we performed IF experiment in cell line. The result (Response New Figure 3, Fig.3A and B) showed that Notch receptor Notch1 and the Notch ligand Dll4 were almost not together in the same endosome. In addition, the result showed that Notch1 barely colocalized with Dll4. This result may be due to the character of the Notch1 antibody we used. This Notch1 antibody specifically recognizes the Notch transmembrane subunit (NTM) region, which consists of a short extracellular juxtamembrane peptide, a transmembrane sequence and the intracellular domain (NICD). The Notch ligand mainly binds with extracellular Notch subunit (ECN) but not NTM. The early endosome localization of NTM is a required step for NICD release and Notch signal activation in normal circumstances (1).

Response New Figure 3 (A) 293T cells were immunostained with antibodies against endogenous NOTCH1 (green), EEA1 (red) and DLL4 (blue). The nucleus was stained with Hoechst and exhibited in white colour. Scale bar, 10μm. (B) Quantification of co-localization of target proteins using Manders’ coefficient (ImageJ). n=14 cells. Error bars, mean ± s.d, ****P* < 0.001.

Specific comments

I have some problems to see the co-localization of Notch members with the endosomal components. In Figure 4 but also other figures, colocalization of notch members with the early endosome marker is not obvious. Could the authors show higher magnification/better resolution of the endosomal images?

**Response 5**: Thanks for pointing out this. We have provided images with higher magnification and better resolution in the revised manuscript (see revised manuscript Fig. 4 O-T, Fig. 5 L-O, Fig. 7 K-P).

The authors should clearly specify in the text all the experiments performed in cell lines, since most of the time it is only in the figure legend.

**Response 6**: Thanks for pointing out this. We have added descriptions mentioning that all these experiments were performed in cell lines.

Do the authors suggest that Notch endosomes are only interacting with EEA1 and LAMP1?  Could they show negative controls? they could test colocalization of Notch members with Appl1 and Akt with EEA1. If the authors are correct, those proteins should not co-localize.

**Response 7**: Thanks for this comment. To answer this question, we performed IF experiment in 293T cell line. The result (Response New Figure 4, Fig.4A and B, also see revised manuscript Fig. S5 D and E) showed that the Notch receptor Notch1 barely colocalized with APPL1. In addition, AKT mainly colocalized with APPL1 but not EEA1 (Response New Figure 4, Fig.4C and D, also see revised manuscript Fig. S5 B and C). These results suggest that Notch signal is mainly regulated by endosome labeled by EEA1, and AKT signal is mainly regulated by endosome labeled by APPL1.

Response New Figure 4 (A) 293T cells were immunostained with antibodies against endogenous NOTCH1 (green), EEA1 (red) or APPL1 (red). The nucleus was stained with Hoechst. Scale bar, 10μm. (B) Quantification of co-localization of target proteins using Manders’ coefficient (ImageJ). n=14 cells. Error bars, mean ± s.d, ****P* < 0.001. (C) 293T cells were immunostained with antibodies against endogenous AKT (green), APPL1 (red) or EEA1 (red). The nucleus was stained with Hoechst. Scale bar, 10μm. (D) Quantification of co-localization of target proteins using Manders’ coefficient (ImageJ). n=14 cells. Error bars, mean ± s.d, ****P* < 0.001.

Although authors have checked Wnt and Erk pathways, they cannot exclude that Rab5c is affecting other pathways a part from Notch or Akt/Pi3K. The authors should mention this possibility.

**Response 8**: Thanks for this thoughtful suggestion. In our RNA-seq data (Fig.3A and B in revised manuscript), there are many down-regulated and up-regulated signaling pathways in Rab5c-deficiency embryos. Among these signaling pathways, Notch and AKT signaling in endothelial cells are crucial for HSPC development (9-12). However, other altered signals, such as oxidative phosphorylation, Arp2/3 protein complex, lipoprotein particle, NF-kappa B signaling and tight junction, also exist in the RNA-seq data. These signals are involved in physiological-biological activities of energy metabolism, cytoskeleton remodeling, lipid metabolism, inflammatory and cell-to-cell contact, which may be also involved in the HSPC defect induced by Rab5c-deficiency. Whether these signals are regulated by Rab5c as well as play essential roles in HSPC development awaits further investigation. We have added more discussions to address this question in the revised manuscript (see revised manuscript, Page 17, line 426-434).

It is also surprising the DN and CA phenotype is similar in terms of Notch activation. Morover both constructs have opposite activity for Rab5c but they seem to affect similarly Notch. The authors should discuss this observation.

**Response 9**: Thanks for this thoughtful comment. Rab5c dominant-negative (DN) was generated by a S36N amino acid mutation in the conserved GTP-binding pocket, which affects GTP/GDP affinity and further leads to endocytic trafficking inhibition (13-15). Rab5c DN overexpression resulted in reduced early endosome localization of Notch ligand and receptor (Fig. 4 O-T in revised manuscript). Early endosome localization of Notch is demonstrated to be a required step for activation of Notch in normal circumstances (1, 8). Therefore, the impaired endocytic trafficking of Notch ligand and receptor may be the reason for Notch signaling defect in Rab5c DN overexpression group.

Rab5c CA was generated by a Q81L amino acid mutation in the conserved GTP hydrolysis region, which affects GTPase activity and leads to excessive endocytic trafficking (13-15). Rab5c CA likely led to a special circumstance in which largely increased early endosome localization of Notch (the data in original manuscript, now in revised manuscript Fig. 7K-P) enhanced the endolysosomal trafficking of Notch, further leading to a dramatic reduction of Notch ligands and receptor molecules (the data in original manuscript, now in revised manuscript Fig. S6 J and K). Our results support the view (16) that excessive endocytic trafficking of Notch attenuates the Notch signaling. Therefore, we speculate that the balance of Rab5c-mediated endocytic trafficking of Notch is important for Notch activation in zebrafish. We have added more discussions on this observation in the revised manuscript (see revised manuscript, Page 16, line 404-415).

Reviewer #2: Paper by Heng et al. provides detailed analysis of Rab5c function during hematopoietic development in zebrafish model. This is a high quality manuscript which revealed that Rab5c-mediated endocytic trafficking regulates Notch signaling to ensure appropriate endosome environment for NICD release during HSPC production.   In addition, authors demonstrated that Rab5c activates AKT signaling through interaction with Appl1 adaptor protein which is present in subset of Rab5-positive endosomes. Similar to down-regulation, Rab5c overactivation caused HE specification defect due to ectopic endocytosis of Notch ligands and receptors and increased their transport to endosome-lysosome for degradation. Overall, manuscript established an important role of Rab5c in HSPC development and revealed major molecular mechanisms implicated in Rab5c function in hemogenic endothelium.

I have just few minor comments:

1. Western blots in Fig. 3C, 4A, 4G and 6A shows a single experiment. It would be helpful to provide quantitative analysis of western blot data from several independent experiments.

**Response 10**: Thanks for pointing out this. We performed quantitative analysis of all these western blot data from several independent experiments and presented statistical results in the revised manuscript (see revised manuscript Fig. 4D, 5B,5K,7B).

2.  Spell-out for some abbreviations (WISH and few others) is missing. It would be helpful to spell-out all abbreviations when using for the first time.

**Response 11**: We apologize for missing the full name for some abbreviations. We have added these full names in the revised manuscript.

Reviewer #3: In this manuscript, Heng and colleagues investigate the role of rab5c in HSPC development in zebrafish. Rab5c was previously identified by the same laboratory in a RNA-seq screen from sorted flk1+runx1- vs. flk1+runx1+ cells to be one of the most up-regulated genes in cells of the hemogenic endothelium. In this study, they use both morpholino oligonucleotides and CRISPR-Cas9 to suppress rab5c expression. They then show by extensive data sets that the gene is essential for the specification of HSPCs by endocytic trafficking of Notch and Akt signaling in zebrafish, which importantly provides new insights in how intrinsic signals are orchestrated to execute cell fate transition. This is interesting work, however some points need to be addressed.

Unfortunately, the picture quality for the main part of the manuscript was very low making it sometimes hard to interpret the data, but this is most likely because of downsizing by the system, since figures in the supplementary data are much better. Some questions may arise because of this issue.

**Response 12**: Thanks for pointing out this. We have provided images with higher magnification and better resolution in the revised manuscript (see revised manuscript Fig. 4 O-T, Fig. 5 L-O, Fig. 7 K-P).

Major comments:

It has been previously shown, that Rab5c is a key regulator of trafficking of the CD93/Multimerin/β1-integrin complex in endothelial cell adhesion and migration (Barbera 2019). Furthermore, another recent study demonstrated that another small GTPase of the Ras-related protein family, Rap1b, promotes notch-mediated hemogenic endothelium specification of PLPM cells during HSPC specification by enhancing integrin-mediated cell adhesion. The authors should further investigate and comment on these recent findings in the context of their study.

**Response 13**: Thanks for pointing out this. Both of CD93/Multimerin/β1-integrin complex and Rap1b are associated with β1-integrin activation (2, 3), which has been demonstrated to facilitate Notch signaling during early somitic wave (15-17 hpf) (3, 4). Previous studies showed that heat-shock induction of NICD at 14 hpf can rescue HSPC defects induced by repression of early somitic Notch signaling, however, heat-shock just 2 h later (about 16 hpf) can not (4, 5). In our study, induction of Notch signaling by heat-shock-induced overexpression of NICD at 20 hpf can rescue HSPC defects in Rab5c-deficiency embryos, suggesting that Rab5c regulates Notch during the late endothelial wave (after 20 hpf), but not early somitic wave. Furthermore, we examined the expression pattern of *rab5c* during zebrafish early developmental stage by whole-mount in situ hybridization (WISH). The result (Response New Figure 5, Fig. 5A, also see revised manuscript Fig.S4H; the data in original manuscript, now in revised manuscript Fig. 1*D*, Fig. S1*B*) showed that *rab5c* was almost not expressed at 14 hpf or 16 hpf, and was specifically expressed in endothelial cells at 20 hpf or 26 hpf. Taken together, we propose that Rab5c may not be involved in integrin-mediated somitic wave regulation. We have added more discussions on these recent findings in the revised manuscript (see revised manuscript, Page 15-16, line 391-402).

Response New Figure 5 (A) The expression pattern of *rab5c* during zebrafish early developmental stage by whole-mount in situ hybridization (WISH). Scale bars, 400 μm.

The authors use a construct for somite-specific overexpression of rab5c mRNA lacking the MO binding site by the mylz promoter and are not able to observe a rescue of the HSPCS phenotype after rab5c knockdown. From this they claim that the somitic wave of HSPC specification is not involved in this process. They should also consider performing early NICD induction at 14 hpf and analysis of sclerotome markers to further confirm that this wave is not involved. Additionally, they should discuss in the text why they perform these different experiments here (in regard to the two different waves, somitic and endothelial, during HSPC development).

**Response 14**: To check whether Rab5c-deficiency impairs somitic wave of HSPC specification, we examined the expression of sclerotome markers by WISH. The results (Response New Figure 6, Fig. 6A and B, also see revised manuscript Fig.S3 F and G) showed that expression of *dlc*, *vegfa* and *shh* was not changed in Rab5c-dificiency embryos at 16 hpf, suggesting Rab5c not involved in somitic wave of HSPC specification.

Response New Figure 6 (A) Expression of *dlc*, *vegfa* and *shh* in control and *rab5c* morphants examined by WISH. Scale bars, 400 μm. (B) Statistical analysis of the WISH. Error bars, mean ± s.d, n.s.: non-significant.

To perform early NICD induction at 14 hpf, we utilized *hsp70*-mediated heat-shock (HS) inducible NICD-2a-GFP overexpression system. Strong GFP expression was detected from 2 hours post heat-shock at 14 hpf (Response New Figure 7, Fig. 7A, also see revised manuscript Fig. S4E), to at least 20 hours post heat-shock. Notably, the GFP expression level at 20 or 26 hpf was comparable to that at 16 hpf (Response New Figure 7, Fig.7A, also see revised manuscript Fig. S4E). The WISH results showed that the HSPC defect in Rab5c-dificiency embryos could be rescued by the heat-shock induction of NICD at 14 hpf (Response New Figure 7, Fig.7B and C, also see revised manuscript Fig. S4 F and G). Although heat-shock induction of Notch at 14 hpf provides a rescue, this NICD induction is sustained throughout the endothelial wave (after 20 hpf). Previous studies showed that heat-shock induction of NICD at 14 hpf can rescue HSPC defects induced by repression of early somitic Notch signaling, however, heat-shock just 2 h later can not (4, 5). Together with that the heat-shock induction of NICD at 20 hpf rescuing the HSPC defect induced by Rab5c-dificiency, we propose that Rab5c regulates HSPC development more likely at the endothelial wave. We will add more discussions in the text to address why we perform these different experiments (see revised manuscript, Page 9, line 208-212).

Response New Figure 7 (A) Fluorescence microscope imaging shows the GFP expression. Scale bar, 400 μm. (B) Expression of *runx1* in control and *rab5c* morphants examined by WISH. Scale bars, 100 μm. (C) Quantification of the *runx1*^+^ cell number. Error bars, mean ± s.d, ****P* < 0.001.

The authors show rab5c expression in endothelial cells at 26 hpf. If it is indeed responsible for notch pathway activation, it should already be present at earlier time-points between 16 and 20 hpf. Please explain/comment.

**Response 15**: Thanks for this question. We examined the expression pattern of *rab5c* during zebrafish early developmental stage. The result (Response New Figure 5, Fig. 5A, also see revised manuscript Fig.S4H; the data in original manuscript, now in revised manuscript Fig. 1*D*, Fig. S1*B*) showed that *rab5c* was almost not expressed at 14 hpf or 16 hpf, and was specifically expressed in endothelial cells at 20 hpf or 26 hpf, suggesting that Rab5c regulates Notch during the late endothelial wave, but not early somitic wave.

Endothelial specific NICD-OE instead of global induction via HS would be better for the Notch rescue experiments in Figure 3 and 6.

**Response 16**: Thanks for this suggestion. We performed endothelial cell (EC)-specific NICD-OE driven by *fli1a* promoter as previously described (6). The WISH results showed that the HSPC defect in Rab5c-dificiency embryos could be rescued by the EC-specific NICD-OE (Response New Figure 8, Fig.8A and B, also see revised manuscript Fig.4 M and N).

Response New Figure 8 (A) Expression of *runx1* in different groups examined by WISH. Scale bars, 100 μm. (B) Quantification of the *runx1*^+^ cell number. Error bars, mean ± s.d, ****P* < 0.001.

In addition, EC-specific NICD-OE also could rescue the HSPC defect induced by Rab5c CA overexpression (Response New Figure 9, Fig.9A and B, also see revised manuscript Fig.7I and J). We have used these new results to replace the HS-NICD data in the main text.

Response New Figure 9 (A) Expression of *runx1* in different groups examined by WISH. Scale bars, 100 μm. (B) Quantification of the *runx1*^+^ cell number. Error bars, mean ± s.d, ****P* < 0.001.

In regard to their apoptosis assays: wouldn't the authors expect to find some double positive (apoptotic and endothelial) cells? What are these apoptotic cells that the authors find in their experiments?

**Response 17**: We used the Tg (*gfi1*:GFP) or Tg (*fli1a*:EGFP) transgenic lines to mark HE or pan-endothelial cells, and performed TUNEL to identify apoptotic cells. The double positive cells in our experiments are apoptotic HE (*gfi1*:GFP) or apoptotic pan-endothelial cells (*fli1a*:EGFP).

Please mention what the numbers below the WISH pictures are. This could be difficult to understand, especially for an unexperienced reader who is not working in zebrafish.

 Please use statistical analyses to investigate whether WISH phenotypes indeed show differences, and quantify immunoblot results.

**Response 18**: Thanks for pointing out this. The numbers below the WISH pictures mean: number of embryos showing representative phenotype / total number of embryos. We added description for that in the revised manuscript. In addition, we performed quantification and statistical analysis of the WISH and immunoblot results (see revised manuscript Fig. 2B, Fig. 2D, Fig. 2E, Fig. 2K, Fig. 2N, Fig. 3C, Fig. 3E, Fig. 3G, Fig. 4D, Fig. 4H, Fig. 4I, Fig. 4L, Fig. 4N, Fig. 5B, Fig. 5F, Fig. 5H, Fig. 5K, Fig. 6C, Fig. 6G, Fig. 6I, Fig. 7B, Fig. 7F, Fig. 7G, Fig. 7J, Fig. S2C, Fig. S4P, Fig. S6K, Fig.S7B, Fig.S7D).

What about other pathways that regulate similar steps of HSPC development (e.g. Wnt, Erk signaling, Shh, Vegf signaling)? How do they relate to the presented pathway?

**Response 19**: The data in our original manuscript showed that Wnt and Erk signalings were not affected, and *flk1* expression was normal (data in original manuscript, now see revised manuscript Fig.S4A, Fig. S5 G-I), in Rab5c-dificiency embryos. We further examined the expression of *shh* and *vegfa* in control and Rab5c deficiency embryos. The WISH results showed that the expression of *shh* and *vegfa* at 16 or 26 hpf was not changed in Rab5c-dificiency embryos compared with control (Response New Figure 6, Fig.6A and B; Response New Figure 10, Fig.10A-C, also see revised manuscript Fig. S3 F and G, Fig.S5 J-L), suggesting that Shh and Vegf signalings might not be involved in Rab5c-dificiency induced HSPC defect. However, the relationship between these signaling pathways in Rab5c-regulated HSPC development awaits further investigation.

Response New Figure 10 (A) Expression of *shh* and *vegfa* in control and *rab5c* morphants examined by WISH. Scale bars, 100 μm. (B) Statistical analysis of the WISH examining *shh*. Error bars, mean ± s.d, n.s.: non-significant. (C) Statistical analysis of the WISH examining *vegfa*. Error bars, mean ± s.d, n.s.: non-significant.

Is it possible to analyze endocytic trafficking in vivo in zebrafish instead of using transfected cells?

**Response 20**: Thanks for this question. We tried to check the localization of Notch ligand and receptor, Eea1 and Appl1 in the endothelium of zebrafish by IF. However, the antibodies, which worked well in human cell lines, did not specifically recognize zebrafish endogenous target proteins (please also see Response New Figure 2, Fig.2A). Besides, in our zebrafish endothelium imaging experiments (such as Fig.2F or Movie S3,S4 in revised manuscript) the maximum magnification was 200×, which could resolve different types of cells but not subcellular organelles or complexes. In comparison, the observation of magnification of 1000× in cell lines was able to resolve subcellular organelles or complexes.

Minor comments:

The AGM region is specific to mammals, the term VDA is more commonly used for zebrafish.

**Response 21**: Thanks for pointing out this. We replaced the description in the revised manuscript.

Please explain the western blot data shown in Supplementary Figure S2B. If wt mRNA was co-injected with the MO, shouldn't there be a rescue of the protein expression and no apparent down-regulation? Is there a specific antibody that can alternatively be used to show that indeed the protein is not there?

**Response 22**: This WT *rab5c* mRNA used in MO effectiveness examination had the *rab5c* MO binding site. The MO could inhibit the translation of exogenous mRNA with its binding site (7). Previously, we performed the rescue experiment by overexpression of *rab5c* mRNA lacking the MO binding site. The western blot result showed that the translation of *rab5c* mRNA lacking the MO binding site was not inhibited by *rab5c* MO injection (Response New Figure 11, Fig.11A and B, also see revised manuscript Fig. S2 E and F).

Response New Figure 11 (A) flag tagged *rab5c* mRNA lacking the *rab5c* MO binding site are co-injected with either control or *rab5c* MO into one-cell stage embryos. The protein level examined by WB. (B) Quantification and statistical analysis of WB. Error bars, mean ± s.d, n.s.: non-significant.

In Supplementary Figure 2SE the mentioned arrows are almost not visible. Please enlarge.

**Response 23**: Thanks for pointing out this. We enlarged these arrows in the revised manuscript.

Why didn’t the authors use their mutants for subsequent analyses?

**Response 24**: In our study, HSPC production was reduced in *rab5c* mutant. However, the HSPC phenotype in *rab5c* mutant was relatively weaker than that in *rab5c* morphants due to genetic compensation. To suppress the genetic compensation, we generated *rab5aa*/*rab5c* or *rab5b*/*rab5c* double mutant*. rab5c* MO-mediated knockdown fully phenocopied the *rab5aa*/*rab5c* or *rab5b*/*rab5c* double mutant. Our study utilized a variety of transgenic lines. Considering that crossing these lines with these double mutants and selecting corresponding mutants by genotyping are very time consuming, we chose to use morphants for detailed analysis subsequently.

Please rearrange Supplementary Figure 2 and 3 to keep the order of appearance of the respective figures in the text.

**Response 25**: Thanks for your suggestion. We rearranged these Figures in the revised manuscript.

What does relatively normal mean for their primitive blood phenotypes in Figure S2C? Are primitive myeloid cells affected?

**Response 26**: In original manuscript, the primitive erythroid cells production was examined and the results showed normal primitive erythroid cells production in Rab5c-deficiency embryos. However, the examination for primitive myeloid cells production was not carried out. Then we further examined the primitive myeloid cells production by WISH. The results showed that the primitive myeloid cells production was also normal in Rab5c-deficiency embryos (Response New Figure 12, Fig.12A and B, also see revised manuscript Fig. S2D). Then we rephrased the statement to claim that primitive hematopoiesis was normal in Rab5c-deficiency embryos.

Response New Figure 12 (A) Expression of *pu.1* in control and *rab5c* morphants examined by WISH. Scale bars, 400 μm. (B) Statistical analysis of the WISH. Error bars, mean ± s.d, n.s.: non-significant.

The phenotype in Figure 2M is rather moderate, please consider rephrasing.

**Response 27**: Thanks for pointing out this. We rephrased the statement in the revised manuscript.

The yellow is hard to read in some of the figures (e.g. Figure 5F), consider reorganising.

**Response 28**: Thanks for pointing out this. To make it easy to read, we changed the color to easily recognized dark yellow.

**References**

1.	Gupta-Rossi N, Six E, LeBail O, Logeat F, Chastagner P, Olry A, et al. Monoubiquitination and endocytosis direct γ-secretase cleavage of activated Notch receptor. The Journal of Cell Biology. 2004;166(1):73-83.

2.	Barbera S, Nardi F, Elia I, Realini G, Lugano R, Santucci A, et al. The small GTPase Rab5c is a key regulator of trafficking of the CD93/Multimerin-2/β1 integrin complex in endothelial cell adhesion and migration. Cell Communication and Signaling. 2019;17(1).

3.	Rho SS, Kobayashi I, Oguri-Nakamura E, Ando K, Fujiwara M, Kamimura N, et al. Rap1b Promotes Notch-Signal-Mediated Hematopoietic Stem Cell Development by Enhancing Integrin-Mediated Cell Adhesion. Dev Cell. 2019;49(5):681-96 e6.

4.	Clements WK, Kim AD, Ong KG, Moore JC, Lawson ND, Traver D. A somitic Wnt16/Notch pathway specifies haematopoietic stem cells. Nature. 2011;474(7350):220-4.

5.	Kim AD, Melick CH, Clements WK, Stachura DL, Distel M, Panakova D, et al. Discrete Notch signaling requirements in the specification of hematopoietic stem cells. EMBO J. 2014;33(20):2363-73.

6.	Liu Z, Tu H, Kang Y, Xue Y, Ma D, Zhao C, et al. Primary cilia regulate hematopoietic stem and progenitor cell specification through Notch signaling in zebrafish. Nat Commun. 2019;10(1):1839.

7.	Zhao BS, Wang X, Beadell AV, Lu Z, Shi H, Kuuspalu A, et al. m6A-dependent maternal mRNA clearance facilitates zebrafish maternal-to-zygotic transition. Nature. 2017;542(7642):475-8.

8.	Itoh M, Kim CH, Palardy G, Oda T, Jiang YJ, Maust D, et al. Mind bomb is a ubiquitin ligase that is essential for efficient activation of Notch signaling by Delta. Dev Cell. 2003;4(1):67-82.

9.	Kanz D, Konantz M, Alghisi E, North TE, Lengerke C. Endothelial-to-hematopoietic transition: Notch-ing vessels into blood. Ann N Y Acad Sci. 2016;1370(1):97-108.

10.	Juntilla MM, Patil VD, Calamito M, Joshi RP, Birnbaum MJ, Koretzky GA. AKT1 and AKT2 maintain hematopoietic stem cell function by regulating reactive oxygen species. Blood. 2010;115(20):4030-8.

11.	Burns CE, Traver D, Mayhall E, Shepard JL, Zon LI. Hematopoietic stem cell fate is established by the Notch-Runx pathway. Genes Dev. 2005;19(19):2331-42.

12.	Lv J, Wang L, Gao Y, Ding YQ, Liu F. 5-hydroxytryptamine synthesized in the aorta-gonad-mesonephros regulates hematopoietic stem and progenitor cell survival. J Exp Med. 2017;214(2):529-45.

13.	Stenmark H, Parton RG, Steele-Mortimer O, Lutcke A, Gruenberg J, Zerial M. Inhibition of rab5 GTPase activity stimulates membrane fusion in endocytosis. EMBO J. 1994;13(6):1287-96.

14.	Clark BS, Winter M, Cohen AR, Link BA. Generation of Rab-based transgenic lines for in vivo studies of endosome biology in zebrafish. Dev Dyn. 2011;240(11):2452-65.

15.	Zhang J, Schulze KL, Hiesinger PR, Suyama K, Wang S, Fish M, et al. Thirty-one flavors of Drosophila rab proteins. Genetics. 2007;176(2):1307-22.

16.	Shaye DD, Greenwald I. Endocytosis-mediated downregulation of LIN-12/Notch upon Ras activation in Caenorhabditis elegans. Nature. 2002;420(6916):686-90.
