# Supplementary material for: Rab5c-mediated endocytic trafficking regulates hematopoietic stem and progenitor cell development via Notch and AKT signaling
Source: PLoS Biol. 2020 Apr 10;18(4):e3000696. doi: 10.1371/journal.pbio.3000696 (PMC7176290; doi:10.1371/journal.pbio.3000696)
Supplement: Attachment — Submitted filename: Response to reviewers_R2.doc [file pbio.3000696.s021.doc]

**Point-by-point response to reviewers**

Reviewer remarks:

Reviewer #1 (Anna Bigas, signed review): The authors have addressed all comments and have considerably improved the manuscript.

**Response 1**: We thank Dr. Anna Bigas for her full support of our revised manuscript.

Reviewer #2: All my critiques are adequately addressed.

**Response 2**: We thank this reviewer for his/her full support of our revised manuscript.

Reviewer #3: The efforts of the authors during the revisions are greatly appreciated. The manuscript has much improved.

I only have to last minor comments:

Page 10 line 233. Fig. 3O-T should be changed to Fig. 4O-T

**Response 3**: Thanks for pointing out this. We have changed the citation order of Fig. 3O-T to Fig. 4O-T in the revised manuscript.

Quantification of western blots should be performed throughout the whole manuscript (e.g. also for S5A S5I).

**Response 4**: Thanks for pointing out this. We performed quantification and statistical analysis of these western blots results (see revised manuscript Fig S5B, S5K).
